# The screening and management of pituitary dysfunction following traumatic brain injury in adults: British Neurotrauma Group guidance

**DOI:** 10.1136/jnnp-2016-315500

**Published:** 2017-08-31

**Authors:** Chin Lik Tan, Seyed Alireza Alavi, Stephanie E Baldeweg, Antonio Belli, Alan Carson, Claire Feeney, Anthony P Goldstone, Richard Greenwood, David K Menon, Helen L Simpson, Andrew A Toogood, Mark Gurnell, Peter J Hutchinson

**Affiliations:** 1 Division of Neurosurgery, Department of Clinical Neurosciences, University of Cambridge, Cambridge Biomedical Campus, Cambridge, CB20QQ, UK; 2 Department of Neurosurgery, Queens Medical Centre, Nottingham, UK; 3 Department of Endocrinology, University College London Hospitals, London, UK; 4 NIHR Surgical Reconstruction and Microbiology Research Centre, Queen Elizabeth Hospital, Birmingham, UK; 5 Centre for Clinical Brain Sciences, University of Edinburgh, Edinburgh, UK; 6 Centre for Neuropsychopharmacology and Computational, Cognitive and Clinical Neuroimaging Laboratory, Division of Brain Sciences, Imperial College London, Hammersmith Hospital, London, UK; 7 Imperial Centre for Endocrinology, Imperial College Healthcare NHS Trust, St Mary’s Hospital, London, UK; 8 Institute of Neurology, University College London, London, UK; 9 Department of Medicine, Division of Anaesthesia, University of Cambridge, Cambridge Biomedical Campus, Cambridge, UK; 10 Department of Endocrinology, Queen Elizabeth Hospital Birmingham, Birmingham, Edgbaston, UK; 11 Wellcome Trust-MRC Institute of Metabolic Science, University of Cambridge, Cambridge Biomedical Campus, Cambridge, United Kingdom

**Keywords:** traumatic brain injury, pituitary dysfunction, screening, management

## Abstract

Pituitary dysfunction is a recognised, but potentially underdiagnosed complication of traumatic brain injury (TBI). Post-traumatic hypopituitarism (PTHP) can have major consequences for patients physically, psychologically, emotionally and socially, leading to reduced quality of life, depression and poor rehabilitation outcome. However, studies on the incidence of PTHP have yielded highly variable findings. The risk factors and pathophysiology of this condition are also not yet fully understood. There is currently no national consensus for the screening and detection of PTHP in patients with TBI, with practice likely varying significantly between centres. In view of this, a guidance development group consisting of expert clinicians involved in the care of patients with TBI, including neurosurgeons, neurologists, neurointensivists and endocrinologists, was convened to formulate national guidance with the aim of facilitating consistency and uniformity in the care of patients with TBI, and ensuring timely detection or exclusion of PTHP where appropriate. This article summarises the current literature on PTHP, and sets out guidance for the screening and management of pituitary dysfunction in adult patients with TBI. It is hoped that future research will lead to more definitive recommendations in the form of guidelines.

## Development of the guidance

### Aims of the guidance

There is currently no widely accepted standard as to which patients should be screened for pituitary dysfunction following a traumatic brain injury (TBI). Several groups outside the UK have provided recommendations,^1–3^ but these lack uniformity with respect to the optimal methods and timing(s) of screening.

This document aims to provide guidance on (1) who, (2) when and (3) how to screen for and manage pituitary dysfunction, including post-traumatic hypopituitarism, in adult patients with TBI, to ensure uniformity of practice across the country. It is primarily targeted at neurosurgeons who deal with TBI on a regular basis, but may also be relevant to clinicians from other specialties, including intensive care, surgery and medicine. It is anticipated that this will also form the basis for future updates of related recommendations when new evidence becomes available.

This document should be used as guidance only, and clinicians must continue to exercise their judgement when deciding on the management plan for individual patients.

### Process of development

A guidance development group comprising expert clinicians involved in the care of patients with TBI, including neurosurgeons, neurologists, neurointensivists and endocrinologists, was convened to develop this guidance. An initial meeting was held in October 2014 at Robinson College, Cambridge, UK. A draft guidance was subsequently disseminated by e-mail to all group members, and revised through multiple iterations to achieve consensus. The final version of the document was approved by all members of the group.

### Levels of evidence and grading of recommendations

The types of evidence and grading of recommendations used in this guidance are in accordance with those set out by the Agency for Healthcare Policy and Research.^4 5^

**Table:** **Types of evidence (adapted from Agency for Healthcare Policy and Research 1992)** 4

Level	Evidence
Ia	Evidence obtained from meta-analysis of randomised controlled trials
Ib	Evidence obtained from at least one randomised controlled trial
IIa	Evidence obtained from at least one well-designed controlled study without randomisation
IIb	Evidence obtained from at least one other type of well-designed quasi-experimental study
III	Evidence obtained from well-designed non-experimental descriptive studies, such as comparative studies, correlation studies and case–control studies
IV	Evidence obtained from expert committee reports or opinions and/or clinical experience of respected authorities

**Table:** **Grading of recommendations (adapted from Agency for Healthcare Policy and Research 1994)** 5

Grade	Evidence levels	Description
A	Ia, Ib	Requires at least one randomised controlled trial as part of the body of literature of overall good quality and consistency addressing the specific recommendation
B	IIa, IIb, III	Requires availability of well-conducted clinical studies but no randomised clinical trials on the topic of recommendation
C	IV	Requires evidence from expert committee reports or opinions and/or clinical experience of respected authorities. Indicates absence of directly applicable studies of good quality

## Introduction

Traumatic brain injury (TBI) has an estimated incidence of 235/100 000 per year based on a systematic review of epidemiological data from 13 European countries,^6^ and results in 300 000 hospitalisations annually in the USA.^7^ In England and Wales, 1.4 million people attend the emergency department with a recent head injury annually, resulting in approximately 200 000 admissions.^8^ TBI most commonly affects young adults and frequently results in devastating consequences, including loss of independence, unemployment and significantly reduced quality of life.

The reported prevalence of post-traumatic hypopituitarism (PTHP) differs significantly between studies. Reasons for this include differences in inclusion/exclusion criteria (eg, severity of TBI), variability in testing protocols (eg, use of static vs dynamic tests), timing(s) of assessment (acute vs later phases) and differing thresholds for diagnosis of hormone deficiencies.^9 10^ A meta-analysis of 14 studies reported the pooled prevalence of PTHP to be 27.5%.^11^ When classified according to the severity of TBI based on the Glasgow Coma Scale (GCS) score, pituitary dysfunction was detected in 16.8% of patients with mild TBI (GCS 13–15), 10.9% with moderate TBI (GCS 9–12) and 35.5% with severe TBI (GCS 3–8).^11^

TBI patients with PTHP may suffer from a variety of clinical sequelae, including physical, psychological and cognitive deficits. Growth hormone (GH) deficiency, which is the most commonly reported endocrine dysfunction following TBI, has been linked to impaired quality of life, depression and poor rehabilitation outcome.^12–14^

### Risk factors

It is not known which groups of patients with TBI are more likely to develop pituitary dysfunction. Various factors have been implicated, but evidence is still lacking.

#### Severity of TBI

Several groups have suggested the severity of TBI, based on the admission GCS score, as a predictor of the development of PTHP (table 1).^15–21^ Kelly *et al* found that a GCS score of <10 was associated with PTHP.^16^ Similarly, in a sample of 104 patients, Klose *et al* reported that 81% of patients who developed PTHP had suffered severe TBI (GCS<9), whereas only 31% of those with normal pituitary function had a history of severe TBI.^19^ A meta-analysis by Schneider and colleagues also demonstrated that the highest rate of PTHP (35.5%) occurred in patients with severe TBI, although the prevalence in moderate TBI (10.9%) was actually lower than that in mild TBI (16.8%).^11^ In contrast, other groups have found no correlation between post-traumatic pituitary dysfunction and the initial GCS score (table 1).^22–27^

**Table 1:** Correlation between TBI severity (GCS score) and prevalence of PTHP

**Study**	**n**	**GCS**	**Time from injury**	**Correlation between TBI severity and PTHP**
Hadjizacharia *et al* 21	436	>12: 41.7% 9–12: 15.8% <9: 42.4%	Mean 1.2 days	Yes
Klose *et al* 20	46	>12: 47.8% 9–12: 19.6% <9: 32.6%	12 months	Yes
Klose *et al* 19	104	>12: 42.3% 9–12: 19.2% <9: 39.4%	Median 13 months	Yes
Bondanelli *et al* 17	50	>12: 32% 9–12: 14% <9: 54%	12–64 months	Yes
Kelly *et al* 16	22	3–15	Median 26 months	Yes
Kozlowski Moreau *et al* 27	55	Lowest GCS average: 8.8	>1 year	No
Schneider *et al* 26	70	3–15	12 months	No
Aimaretti *et al* 25	70	>12: 47.1% 9–12: 31.4% <9: 21.4%	12 months	No
Agha *et al* 23	102	9–12: 44.1% <9: 55.9%	Median 17 months	No
Lieberman *et al* 22	70	–	Median 13 months	No

GCS, Glasgow Coma Scale; PTHP, post-traumatic hypopituitarism; TBI, traumatic brain injury.

#### Imaging findings

Kelly *et al* reported that certain imaging findings, such as diffuse brain swelling, on initial CT head scan predicted PTHP,^16^ while Schneider *et al* found an association between PTHP and diffuse axonal injury or basal skull fracture.^26^ However, two other studies failed to identify a convincing association between PTHP and brain imaging findings, including intracranial haematomas and diffuse axonal injury.^17 23^

#### Other factors

Raised intracranial pressure (ICP) has been shown to be associated with PTHP.^28 29^ Other factors implicated as predisposing to PTHP include hypotension and hypoxia,^16^ older age^26^ and prolonged intensive care unit stay.^28^ In contrast, possession of apolipoprotein E3/E3 genotype has been linked to a reduced risk of hypopituitarism following TBI.^30^

Blast TBI may be a particular risk factor for pituitary dysfunction.^31^ Thirty-two per cent of soldiers after moderate-severe blast TBI (defined according to the Mayo classification)^32^ developed anterior pituitary dysfunction, compared with 2.6% of age-matched and gender-matched civilians after moderate-severe non-blast TBI.^31^

### Pathophysiology

While the exact mechanisms underlying pituitary dysfunction have not been elucidated, several hypotheses have been proposed, with Dusick and colleagues concluding that it is likely the result of a combination of pathological processes.^33^

The most widely accepted theory is that of an ischaemic insult to the pituitary gland.^33 34^ TBI can be associated with direct damage to the hypophyseal portal veins,^35 36^ direct trauma to the gland^37 38^ or transection of the pituitary stalk.^35 39^ These, in association with the hypotension, hypoxia and brain swelling that frequently accompany TBI, lead to pituitary ischaemia and infarction.^40^ This is supported by postmortem findings in patients with TBI where the pathological features include capsular haemorrhage around the pituitary (59%), posterior lobe haemorrhage (31%), anterior lobe necrosis (22%) and stalk necrosis (3%).^36 41^ In addition, a significant proportion of patients (42%) have been reported to manifest ischaemic/haemorrhagic lesions of the hypothalamus.^37^

Several other factors have also been implicated in the pathogenesis of PTHP. The pituitary gland is susceptible to direct mechanical impact in TBI, particularly in those with a base of skull fracture. Acceleration/deceleration forces may cause shearing of white matter tracts connected to the pituitary gland. It has been suggested that GH and gonadotrophin deficiency are most commonly seen post-TBI due to the more laterally located somatotrophs and gonadotrophs.^42 43^

In vivo, imaging techniques in patients with moderate-to-severe TBI have demonstrated pituitary enlargement in the acute phase.^39^ Over time, patients with TBI may exhibit loss of pituitary volume or an empty sella, abnormal enhancement, perfusion deficits and/or lack of the posterior pituitary signal.^44^ Furthermore, 30% of patients show focal changes, including (1) haemorrhage/haemorrhagic infarction, (2) a swollen gland with bulging superior margin, (3) heterogeneous signal in the anterior lobe and (4) partial transection of the infundibular stalk, following TBI.^39^ Soldiers after blast TBI who have pituitary dysfunction display more severe white matter tract damage as assessed by diffusion tensor imaging than those who have an intact hypothalamic–pituitary axis.^31^

Autoimmune-mediated pituitary dysfunction has also been postulated: higher titres of antipituitary (APAs) and antihypothalamic (AHA) antibodies were detected in patients with TBI compared with controls at 3 and 5 years after the initial insult.^45 46^ Boxers who suffer from chronic, repetitive insults to their brain also commonly have detectable circulating AHA (21.3%) and APA (22.9%). Furthermore, 46.2% of AHA-positive boxers have pituitary dysfunction, compared with just 10.4% in AHA-negative subjects.^45 47^

### Hypopituitarism

#### Anterior pituitary dysfunction

##### Acute phase

In the days following TBI, the prevalence of PTHP has been suggested to be as high as 53%–78% (table 2a). GH and gonadotrophins (luteinising hormone (LH) and follicle-stimulating hormone (FSH)) appear to be most commonly affected, and in the first 24 hours after TBI, approximately 20%–30% of patients were reported to manifest abnormalities in serum GH and insulin-like growth factor 1 (IGF-1) concentrations^48 49^; however, these observations were based on basal (non-dynamic) measurements, and therefore cannot be considered diagnostic of GH deficiency (GHD). Two recent studies have suggested high rates (52%–78%) of hypothalamic–pituitary–adrenal axis dysfunction within the first 10 days after injury, based on early morning (09:00) thresholds for (total) plasma/serum cortisol  of <276 nmol/L and <300 nmol/L, respectively.^48 50^ In a cohort of 58 patients admitted to the neurocritical care unit following TBI, 10.3% had abnormalities of the pituitary–adrenal axis within 7 days.^51^ Accepting that the primary endocrine task in acute TBI is to identify those patients with acute hypocortisolism who are at risk of developing life-threatening complications (eg, inotrope-resistant hypotension, hypoglycaemia and hyponatraemia), these findings would suggest a high rate of requirement for exogenous glucocorticoid therapy. However, it is noteworthy that while there was a clear association between acute hypocortisolism and mortality in the study of Hannon and colleagues, those individuals in the lowest quartile for plasma cortisol had the highest mortality despite empirical treatment with hydrocortisone. In addition, no patients in the study of Olivecrona *et al* were given glucocorticoids, and yet no association was observed between low cortisol levels and increased mortality or unfavourable outcome.^48 50^ Accordingly, it remains unclear whether using simple numerical thresholds to diagnose hypoadrenalism in acute TBI facilitates earlier detection of potentially life-threatening hypoadrenalism or leads to unnecessary glucocorticoid therapy.

**Table 2a:** Prevalence of anterior hypopituitarism following TBI (<1 month)

**Study**	**n**	**GCS**	**Time from injury**	**Anterior hypopituitarism (axis affected)**					
				**Total (%)**	**GH (%)**	**FSH/LH (%)**	**ACTH (%)**	**TSH (%)**	**Multiple (%)**
Alavi *et al* 51	58	<14	0–7 days	10.3	–	–	10.3	–	–
Hannon *et al* 50	100	<14	1–10 days	78	–	–	78	–	–
Olivecrona *et al* 48*	45	<9	1 day	–	30.2	55.2	52.3–54.5	9.1	–
			4 days	–	2.3	58.6	59.1–70.5	27.3	–
Krahulik *et al* 66	186	3–14	Acute	53	37	33	10	3	–
Klose *et al* 20	46	3–15	0–12 days	76	–	67	4	33	28
Tanriverdi *et al* 49	52	3–15	24 hours	56	20	41	9.8	6	–
Agha *et al* 52	50	8–13	Acute	–	18	80	16	2	–

*Serum cortisol levels were measured in the morning and in the evening.

ACTH, adrenocorticotropic hormone; FSH, follicle-stimulating hormone; GCS, Glasgow Coma Scale; GH, growth hormone; LH, luteinising hormone; TBI, traumatic brain injury; TSH, thyroid-stimulating hormone.

**Table 2b:** Prevalence of anterior hypopituitarism following TBI (<12 months)

**Study**	**n**	**GCS**	**Time from injury**	**Anterior hypopituitarism (axis affected)**					
				**Total (%)**	**GH (%)**	**FSH/LH (%)**	**ACTH (%)**	**TSH (%)**	**Multiple (%)**
Abadi *et al* 67	75	9–13	3 months	48	24	16	13	5.3	13
			6 months	–	9.3	10.7	4	2.7	–
Schneider *et al* 68*	825	–	<5 months	17–39	22	–	–	–	–
Krahulik *et al* 66	186	3–14	3 months	22.9	–	–	–	–	–
			6 months	18.2	–	–	–	–	–
Klose *et al* 20	46	3–15	3 months	13	–	–	–	–	6.5
Schneider *et al* 69	78	3–15	3 months	56	9	32	19	8	–
Agha *et al* 56	48	8–13	6 months	–	12.5	22.9	18.8	2.1	–
Aimaretti *et al* 24	100	3–15	3 months	35	37	17	8	5	–

*Patients were categorised into acute (<5 months) or chronic (>5 months) groups in this study.

ACTH, adrenocorticotropic hormone; FSH, follicle-stimulating hormone; GCS, Glasgow Coma Scale; GH, growth hormone; LH, luteinising hormone; TBI, traumatic brain injury; TSH, thyroid-stimulating hormone.

**Table 2c:** Prevalence of anterior hypopituitarism following TBI (≥12 months)

**Study**	**n**	**GCS**	**Time from injury**	**Anterior hypopituitarism (axis affected)**					
				**Total (%)**	**GH (%)**	**FSH/LH (%)**	**ACTH (%)**	**TSH (%)**	**Multiple (%)**
Alavi *et al* 51	47	3–15	>6 months*	21.3	–	21.3	4.3	0	12.8
	22	3–15	>12 months	9.1	9.1	–	–	–	–
Kopczak *et al* 70	340	–	<1 month to 39 years	36.5	7.8	40	1.2	5.6	5.6
Hannon *et al* 50	100	<14	Median 14 months	34.4	18.8	3.1	18.8	0	3.1
Tanriverdi *et al* 46	25	3–15	1 year	48	44	8	16	4	16
			3 years	23.5	23	0	0	0	0
			5 years	32	28	4	1	0	1
Ulfarsson *et al* 71	51	<9	2–10 years	27.5	21.6	3.9	0	2	–
Kozlowski Moreau *et al* 27	55	Lowest GCS average 8.8	>1 year	76.4	63.6	3.6	27.3	21.8	–
Kokshoorn *et al* 72	112	–	Mean 4 years	5.4	2.7	0.9	1.8	–	–
Schneider *et al* 68 ^†^	825	–	>5 months	37–38	–	–	–	–	–
Berg *et al* 73	246	<13	Average 12 months	21	5	9	1	12	3
Krahulik *et al* 66	186	3–14	12 months	21	13.5	5.6	–	–	–
Kleindienst *et al* 74	71	3–15	24–36 months	–	35	0	61	0	–
Wachter *et al* 40	55	3–15	1–4 years	25.4	1.8	12.7	3.6	1.8	1.8
Tanriverdi *et al* 75	30	3–15	1 year	–	43.3	3.3	20	6.6	–
			3 years	–	23.3	0	6.6	0	–
Klose *et al* 19	104	3–15	Median 13 months	15	15	2	5	2	3.8
Klose *et al* 20	46	3–15	12 months	10.9	10.9	2.1	6.5	2.1	6.5
Herrmann *et al* 76	76	<8	Median 20 months	24	8	17	2	2	6.6
Schneider *et al* 69	78	3–15	12 months	36	10	20	9	3	4.3
Tanriverdi *et al* 49	52	3–15	12 months	59	32	7.7	19	6	9.6
Leal-Cerro *et al* 77	170	<8	>12 months	24.7	5.8	17	6.4	5.8	8.8
Aimaretti *et al* 25	70	3–15	12 months	22.7	18.6	11.4	7.1	5.7	10.0
Agha *et al* 23	102	3–13	Median 17 months	28	10.7	11.8	12.7	1	5.9
Agha *et al* 52	50	8–13	12 months	–	10.4	12.5	18.8	2.1	–
Bondanelli *et al* 17	50	3–15	12–64 months	54	28	14	0	10	12
Popovic *et al* 12	67	9–13	Median 44 months	34	15	9	7	4	10.4
Lieberman *et al* 22	70	–	Median 13 months	68.5	14.6	1.4	45.7	21.7	–
Kelly *et al* 16	22	3–15	Median 26 months	36.4	18.2	22.7	4.5	4.5	–

* Patients were assessed at >6 months in this study.

†Patients were categorised into acute (<5 months) or chronic (>5 months) groups in this study.

ACTH, adrenocorticotropic hormone; FSH, follicle-stimulating hormone; GCS, Glasgow Coma Scale; GH, growth hormone; LH, luteinising hormone; TBI, traumatic brain injury; TSH, thyroid-stimulating hormone.

It is also important to exercise caution when interpreting the results of endocrine testing during the acute phase of TBI because of the well-recognised hormonal changes that occur in normal subjects in response to acute intercurrent (non-endocrine) illness (eg, apparent central hypothyroidism due to non-thyroidal illness, for which there is little evidence of a role for thyroid hormone replacement therapy). It is conceivable therefore that at least some of the temporary hormone deficits noted in acutely ill patients, which resolve on follow-up testing, reflect a normal adaptive endocrine response rather than transient hypopituitarism.

##### Chronic phase

Disturbance of normal pituitary function appears to be transient in many patients, with rates of PTHP declining over time (tables 2a–c), although again with marked variation between studies (eg, 13%–56% at 3–6 months, 5.4%–76.4% at >12 months after TBI). Similarly, the extent of pituitary dysfunction differs markedly (tables 2a–c) and again reflects, at least in part, the variation in methodologies used and diagnostic tests employed. Nevertheless, GHD and hypogonadotropic hypogonadism remain the two most commonly identified endocrine deficits (similar to the acute phase), while most studies have found that <10% of patients manifest abnormalities of the ACTH-adrenal and thyroid-stimulating hormone (TSH)-thyroid axes in the longer term. Alavi *et al* found that of the 22 patients assessed at >12 months following TBI, only two (9.1%) manifested GHD.^51^ One group who followed their patients at 1 year, 3 years and 5 years post-TBI demonstrated a progressively decreasing trend for pituitary deficits from the first year to the fifth.^46^ In a study of 48 patients by Agha and colleagues, none of the 14 subjects who had normal anterior pituitary function when tested at 6 months, including dynamic endocrine tests, had developed PTHP at 12 months.^52^ Furthermore, 7 of the 34 subjects who had pituitary dysfunction at 6 months had recovered by 12 months.^52^ Recently, Cuesta *et al* identified symptoms of gonadal dysfunction as being the most reliable predictors of hypopituitarism in patients >6 months post-TBI, while in contrast non-specific symptoms were no more predictive than unselected screening.^53^

As already highlighted, much of the variation in prevalence rates can likely be attributed to differences in patient selection, investigations employed and thresholds applied to diagnose endocrine insufficiency.^9 10^ This is supported by the findings of a study that directly compared the use of different tests and cut-offs in determining the prevalence of post-traumatic GHD, and found significant discrepancies within the same individuals.^29^ Specifically, the prevalence of GHD was lower when using local, compared with guideline-defined cut-offs, while insulin tolerance testing yielded a lower prevalence of GHD compared with the pyridostigmine-growth hormone-releasing hormone (PD-GHRH) or GHRH-arginine test.^29^

#### Posterior pituitary dysfunction

Posterior pituitary dysfunction following TBI is typically transient (<1 month duration). In the acute phase, the proportion of patients developing cranial diabetes insipidus (DI) again varies markedly between studies, ranging from 2.6% to 51% (table 3),^21 50 54–57^ and perhaps reflecting the inherent difficulties in establishing a firm diagnosis in the presence of confounding factors (eg, vigorous fluid resuscitation, use of hypertonic fluids, etc). Follow-up data indicate that few patients require long-term desmopressin therapy: Aimaretti *et al* reassessed 100 patients with TBI at 3 months’ postinjury and found that only 4% had DI^24^; at 6–9 months, 12 months and 17 months, the percentages of patients with persistent DI were 1.4%–8%,^57 58^ 6%^57^ and 6.9%,^55^ respectively (table 3). Three studies have reported that the development of DI is associated with increased mortality.^21 50 54^

**Table 3:** Prevalence of posterior pituitary dysfunction following TBI

**Study**	**n**	**GCS**	**Time from injury**	**% of posterior hypopituitarism (cranial diabetes insipidus)**
**<1** month				
Hannon *et al* 50	100	<14	Median 6 days	51
Hadjizacharia *et al* 21	436	3–15	Mean 1.2 days	15
Agha *et al* 57	50	<14	1–3 days	26
Agha *et al* 55	102	<14	Acute	21.6
Agha *et al* 56	50	<14	Median 12 days	26
Boughey *et al* 54	–	–	Mean 1.5 days	2.9
**>1** month				
Bavisetty *et al* 58	70	Median 7	6–9 months	1.4
Agha *et al* 57	50	<14	6 months	8
			12 months	6
Agha *et al* 55	102	<14	Median 17 months	6.9
Aimaretti *et al* 24	100	3–15	3 months	4

GCS, Glasgow Coma Scale; TBI, traumatic brain injury.

The syndrome of inappropriate antidiuretic hormone (SIADH) is also well recognised in the context of TBI. Hyponatraemia is most commonly seen within the first week after injury and may be multifactorial. The incidence of SIADH varies from 2.3% to 36.6%, although recent studies suggest that between 1 in 7 and 1 in 8 patients are affected.^55 56 59^ In most cases, it is a transient phenomenon: Agha *et al* found that of the 13 patients who had SIADH in the acute phase, only one persisted into the chronic phase.^55^

## Guidance

To screen all patients with TBI for pituitary dysfunction would represent a major logistical challenge and would likely yield false-positive results (especially in the acute phase due to physiological changes associated with intercurrent illness), leading to unnecessary treatment in some patients. However, given the potentially serious clinical consequences of failing to diagnose hypopituitarism (especially hypoadrenalism), there is a pressing need to more reliably identify those patients with TBI who are at greatest risk.

### Anterior pituitary function testing during hospital admission

Some authors advise screening for hypocortisolism (relative or absolute) as soon as possible in all patients with clinical features and/or risk factors for acute adrenal insufficiency.^60 61^ Glynn and Agha have recommended that serum cortisol levels are monitored in the first 7 days post-TBI, so that glucocorticoid replacement can be initiated if there is any suspicion of glucocorticoid deficiency.^10^ For patients with mild TBI, Tanriverdi and colleagues proposed that screening for hypopituitarism (specifically ACTH and TSH deficiency) should be undertaken if (1) the patient is expected to be hospitalised for >24 hours; (2) CT head shows an abnormality (brain swelling, diffuse axonal injury, basal skull fracture, epidural/subdural haematoma, cranial vault fractures); and (3) the patient manifests signs or symptoms of hypopituitarism.^61^ Most authors agree that acutely unwell patients with suspected hypoadrenalism should be treated urgently with parenteral high-dose glucocorticoids (see below). In contrast, in stable patients with central hypoadrenalism, lower dosages of oral hydrocortisone (15–25 mg/day) or prednisolone (2.5–5 mg/day) may suffice.^50 62^

However, as discussed above, given the challenges inherent in interpreting the results of endocrine tests in acutely ill patients, and the discordant findings in studies of cortisol replacement therapy based on numerical thresholds, we do not recommend routine testing of pituitary function or measurement of serum/plasma cortisol levels in the acute phase after TBI. If there is a clinical suspicion of cortisol insufficiency, for example, hypotension (especially pressor-resistant) or hyponatraemia, empirical treatment with hydrocortisone 50mg 6–8 hourly, intravenously or intramuscularly; or 50–100mg as an initial intravenous bolus followed by an infusion of 4–8mg/hour should be given immediately after taking a serum/plasma sample for random cortisol measurement. A discussion with the endocrinology team should follow within 24–48 hours. We recognise that some patients may be given glucocorticoid therapy for reasons other than suspected classical cortisol deficiency in the acute phase. Most of these patients will wean from therapy uneventfully, but where there is difficulty with withdrawing glucocorticoids we recommend assessment by an endocrinologist.

#### Recommendations

**Figure 1:**
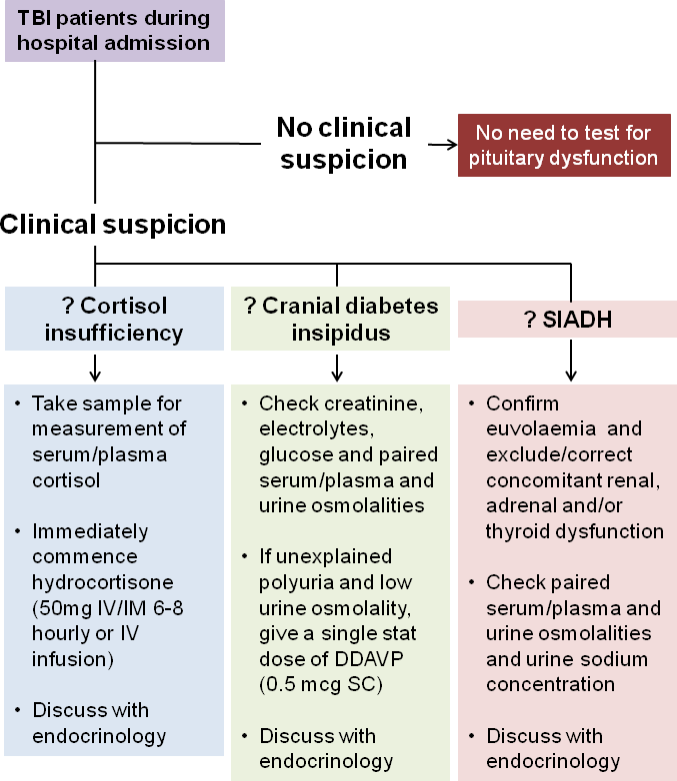
Recommendations for screening for pituitary dysfunction in the acute phase of TBI. SIADH, syndrome of inappropriate antidiuretic hormone; TBI, traumatic brain injury.

- Early (acute phase) testing for pituitary dysfunction is not recommended for the purpose of detecting post-TBI hypopituitarism. (IV, C)
- Routine measurement of plasma/serum cortisol levels is not indicated in the acute phase following TBI. (IV, C)
- If there is clinical suspicion of cortisol insufficiency (eg, refractory hypotension, hypoglycaemia, hyponatraemia), immediately start empirical replacement with hydrocortisone 50mg 6-8 hourly, intravenously or intramuscularly; or 50-100mg as an initial intravenous bolus followed by an infusion of 4-8mg/hour after taking a serum/plasma sample for random cortisol measurement, and discuss with endocrinology. (IV, C)
- If a patient is started on glucocorticoid therapy for reasons other than classical cortisol deficiency, and there is subsequent difficulty in withdrawing treatment, discuss with endocrinology. (IV, C) (figure 1)

### Posterior pituitary function testing during hospital admission

Cranial DI has been linked with increased mortality following TBI, and is a predictor of other long-term pituitary deficits.^50^ Therefore, early recognition of cranial DI in patients with TBI displaying hypernatraemia and hypotonic polyuria is important.^1 10^ Tritos *et al* advise an on-demand approach to desmopressin replacement in this context, with careful monitoring of fluid balance and serum sodium levels, as DI is often transient.^62^

We recommend that if cranial DI is suspected, serum creatinine, electrolytes, plasma glucose and paired serum/plasma and urine osmolalities should be checked. Treatment with a single stat dose of desmopressin 0.5 μg subcutaneously should be given if there is unexplained polyuria and low urine osmolality. Early endocrine review is advised. Regular desmopressin orally or nasally may be required for a period of time. However, it is important to note that in patients with raised ICP, hypernatraemia may be a protective mechanism, and permissive hypernatraemia is sometimes used to treat intractable raised ICP, particularly in the intensive care setting. Thus, desmopressin should be used judiciously in patients with raised ICP.

SIADH may complicate TBI, and in the presence of hyponatraemia, water overload, cortisol insufficiency and hypothyroidism should be considered as potential contributory factors. If there is suspicion of SIADH, euvolaemia should be confirmed, along with investigations to check paired serum/plasma and urine osmolalities and urine sodium concentration. Renal, adrenal and/or thyroid dysfunction should be excluded or corrected. An endocrinology review should follow. It is beyond the scope of this guidance to discuss the different strategies that may be required to diagnose and treat hyponatraemia, but the reader is directed to recently published comprehensive guidelines.^63^

#### Recommendations

- Cranial DI should be considered at an early stage in patients with TBI displaying hypernatraemia and hypotonic polyuria. (III, B)
- If cranial DI is suspected, check serum creatinine, electrolytes, plasma glucose and paired serum/plasma and urine osmolalities, give a stat dose of desmopressin 0.5 μg subcutaneously, and discuss with endocrinology. (IV, C)
- Although transient DI is recognised following TBI, some patients will need to be established on regular desmopressin (either orally or nasally) if there is unexplained polyuria and low urine osmolality, with subsequent reappraisal of ongoing long-term requirements by an endocrinologist. (IV, C)
- If SIADH is suspected in a patient with hyponatraemia, confirm euvolaemia, exclude/correct renal, adrenal and/or thyroid dysfunction, and check paired serum/plasma and urine osmolalities and urine sodium concentration, and discuss with endocrinology. (IV, C) (figure 1)

### Screening for pituitary dysfunction following discharge from hospital

Although deficiencies in GH, FSH/LH and to a lesser extent TSH are likely to be of limited significance in the acute phase following TBI, in the longer term they may result in significant morbidity if left undiagnosed/untreated. Due to the potential confounding effects of non-thyroidal illness (sick euthyroid syndrome) and the long half-life of thyroxine, it has been suggested that screening for hypothalamic–pituitary–thyroid dysfunction can reasonably be deferred for several (>4–6) weeks after TBI (assuming there is no suspicion of prior thyroid dysfunction).^50 62^ Some authors recommend screening for pituitary dysfunction at 3–6 months in all patients with moderate to severe TBI, using a combination of basal (non-stimulated) and, where appropriate, dynamic tests.^1 10 11 64 65^ Glynn and Agha have recommended screening for GH, gonadal, adrenal and thyroid dysfunction at 3–6 months, and that any hormone deficiencies identified at this stage should be replaced, with a view to repeat assessment at 1 year.^10^

**Figure 2:**
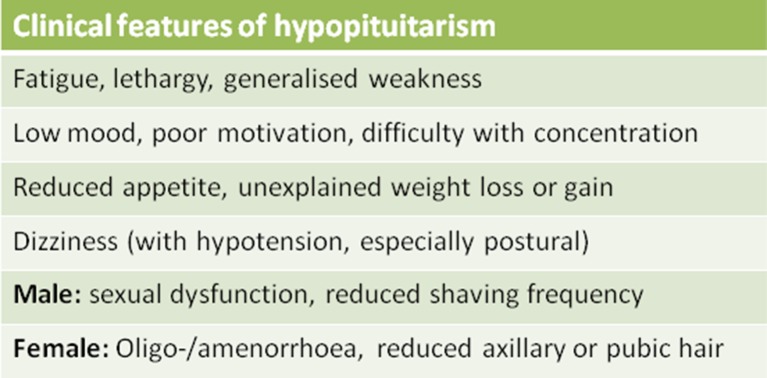
Symptoms suggestive of pituitary dysfunction.

Based on the available evidence, anyone admitted for more than 48 hours for the management of TBI should undergo screening for pituitary dysfunction at 3–6 months using the tests shown in box 1. Patients with abnormal screening test results at 3–6 months should be referred to endocrinology for a full assessment that may include dynamic testing for GHD. Patients who are not admitted, or who are admitted for less than 48 hours, may require screening for pituitary dysfunction if they experience ongoing symptoms consistent with hypopituitarism. These symptoms can include fatigue, low mood, poor motivation, reduced appetite, loss of libido,  sexual dysfunction (in men) and oligomenorrhoea/amenorrhoea (in women) (figure 2). Additionally, other causes of these symptoms should also be considered (including depression) and should be screened for using a standard brief screening tool such as the Hospital Anxiety and Depression (HAD) scale, Beck Depression Inventory II (BDI-II) or Patient Health Questionnaire (PHQ-9). It is important to bear in mind that there may be considerable overlap between the symptoms associated with TBI itself (particularly as a result of diffuse axonal injury) and those of pituitary dysfunction, which may exacerbate each other. Symptoms can therefore have a poor specificity for the presence of pituitary dysfunction.

Box 1 **Screening for pituitary dysfunction post-traumatic brain injury (post-TBI)**

The following tests (serum/plasma sample)* should be performed at 09:00:

**Men**

Urea, creatinine and electrolytes

Free T4 and thyroid-stimulating hormone (TSH)

Cortisol

Luteinising hormone (LH), follicle-stimulating hormone (FSH), testosterone, sex hormone-binding globulin, albumin

**Women**

Urea, creatinine and electrolytes

Free T4 and TSH

Cortisol† and

**In premenopausal women**

If menstrual cycle has become abnormal post-TBI, check LH, FSH, oestradiol

**In postmenopausal women**

FSH

*If in doubt, check local laboratory requirements.†If the patient is taking an oestrogen-containing oral contraceptive pill or oral hormone replacement therapy, we recommend seeking endocrine advice prior to assessing hypothalamic–pituitary–adrenal axis function.

If cortisol insufficiency (09:00 serum/plasma cortisol <100 nmol/L) is found, the patient should be started on oral hydrocortisone immediately (10 mg on waking, 5 mg at noon, 5 mg at 17:00) and referred to endocrinology on an urgent basis. If the cortisol level is 100–400 nmol/L, a referral for further assessment should be made and the patient advised to follow ‘steroid sick day rules’ with emergency hydrocortisone cover until they have been reviewed by an endocrinologist. Referral to endocrinology for assessment of adrenal status is generally not required if the serum/plasma cortisol level is more than 400 nmol/L.

Note that while a serum/plasma IGF-I level below the reference range for age and gender is suggestive of GHD, it is not diagnostic, and requires referral to an endocrinologist for dynamic testing to investigate for GHD and coincident ACTH/cortisol deficiency. Similarly, a serum/plasma IGF-I within the age-related reference range does not exclude GHD. However, referral for dynamic testing of GHD in a subject with an IGF-I level in the normal range can, in most instances, be deferred to >12 months after TBI, since GH replacement would generally not be considered until this time point as spontaneous recovery may occur in some subjects. If the patient remains symptomatic at this stage, then formal testing for GHD is indicated.

#### Recommendations

**Figure 3:**
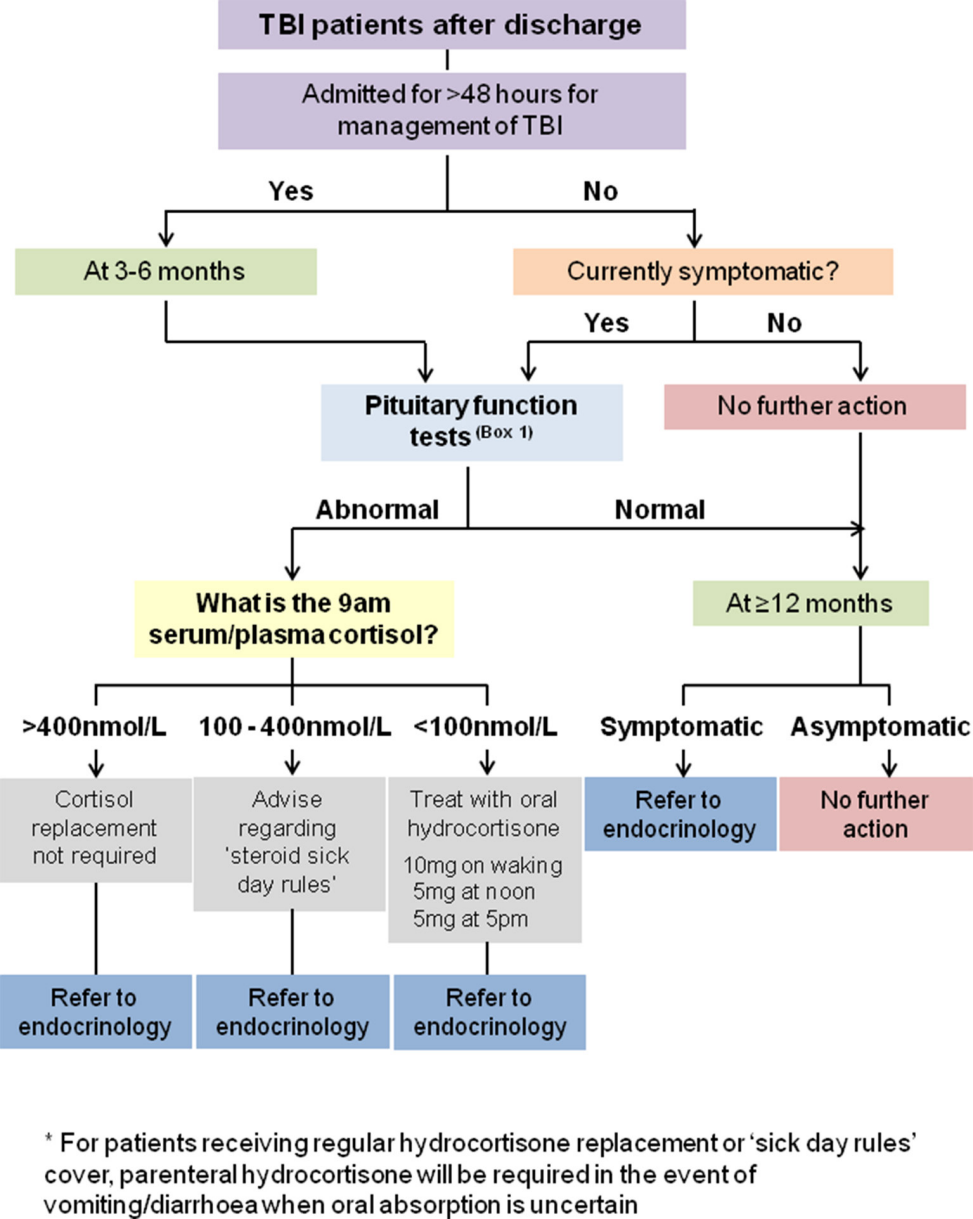
Recommendations for screening for pituitary dysfunction in patients with traumatic brain injury (TBI) following discharge.

- Patients requiring admission for >48 hours following TBI should undergo screening at 3–6 months to exclude pituitary dysfunction (box 1). (IV, C)
- Patients with abnormal screening test results at 3–6 months or at 12 months should be referred to endocrinology for a full assessment. (IV, C)
- Patients not admitted, or admitted for less than 48 hours, but who have ongoing symptoms potentially consistent with pituitary dysfunction may warrant screening for pituitary dysfunction (box 1). (IV, C)
- Patients with ongoing symptoms of pituitary dysfunction (as above, box 1) should also be screened for depression using a standard brief screening tool such as the HAD scale, BDI-II or PHQ-9. (IV, C)
- The following ‘action limits’ for 09:00 serum/plasma cortisol are advised: <100 nmol/L, start treatment with oral hydrocortisone (10 mg on waking, 5 mg at noon and 5 mg at 17:00) immediately and refer urgently to endocrinology; 100-400 nmol/L, refer to endocrinology and advise adherence to ‘steroid sick day rules’ with emergency hydrocortisone cover (until review by endocrinology); >400 nmol/L, referral to endocrinology is not routinely required. (IV, C) (figure 3)

### More than 1 year after TBI

Screening for GHD within a year of TBI is probably not necessary as it may be temporary. However, patients with a history of TBI who continue to have or develop symptoms of possible hypopituitarism beyond 12 months should be referred to endocrinology for consideration of testing for GHD and other pituitary dysfunction.

#### Recommendation

- Patients with a history of TBI who continue to display or develop symptoms of possible hypopituitarism beyond 12 months should be referred to endocrinology for consideration of testing for GH and other pituitary deficiencies. (III, B) (figure 3)

## Future research

The many uncertainties surrounding PTHP are related to the relatively small number of studies in this area. Further research is warranted, and we believe the following areas deserve particular attention:

- What is the prevalence of pituitary dysfunction in mild TBI and persistent postconcussion syndrome?
- What is the prevalence of late-onset (>12 months) pituitary dysfunction post-TBI?
- Is there a demographic, clinical and/or imaging phenotype that reliably predicts those at particular risk of pituitary dysfunction?
- What is the hormone signature during the first 48 hours post-TBI?
- What are the pathophysiological mechanisms underlying pituitary dysfunction post-TBI?
- What is the impact of GH replacement on the quality of life and neurocognitive function after TBI in those who have GHD, and how does it relate to long-term sequelae?
- What is the health economic analysis of pituitary function testing after TBI?

## Conclusion

There remain significant challenges in relation to the diagnosis, investigation and management of PTHP. First, TBI is common, and it would not be feasible (both on capacity and economic grounds), or indeed necessary (for the reasons outlined above), to perform endocrine testing in every patient with TBI. Despite the publication of several case series, the incidence of PTHP remains a matter of considerable debate with divergent rates reported between centres. In addition, although some data suggest that severe TBI is a particular risk factor for PTHP, even in this subgroup it is not clear which patients are at greatest risk and should be prioritised for investigation and treatment. The situation is further confounded by the considerable overlap in symptoms following TBI in patients with and without pituitary dysfunction. Finally, while single (static) blood tests may be used for screening purposes, the limitations of such an approach are well recognised, and definitive investigation frequently requires more complex dynamic hormone testing with interpretation by an experienced endocrinologist.

We therefore recommend a pragmatic approach to screening for PTHP as set out above, but acknowledge there remains controversy surrounding whom to test, when to test and how to test. We suggest that there is an urgent need for a multicentre study powered to answer questions regarding the true incidence of PTHP, to determine the clinical and imaging parameters that predict which patients are at greatest risk, and to identify the optimal methods for, and timing(s) of, endocrine investigation.
